# Tenosynovial giant cell tumor of the posterior pharyngeal space

**DOI:** 10.1002/ccr3.5351

**Published:** 2022-02-06

**Authors:** Alexandros Poutoglidis, Nikolaos Tsetsos, Vasileios Chatzinakis, Christos Georgalas

**Affiliations:** ^1^ Department of Otorhinolaryngology‐Head and Neck Surgery "G. Papanikolaou" General Hospital Thessaloniki Greece; ^2^ Endoscopic Skull Base Centre Hygeia Hospital Athens Greece; ^3^ Medical School University of Nicosia Nicosia Cyprus

**Keywords:** giant cell tumor, oncology, pharyngeal space, pharyngeal wall tumor

## Abstract

A 55‐year‐old man presented with dysphagia and a sore throat. Oral examination revealed a firm nodular mass in the midline of the pharyngeal wall. The tumor was en‐bloc excised. Histopathology and immunohistochemistry confirmed the diagnosis of a tenosynovial giant cell tumor.

## Graphical abstract

Pharyngeal wall tumors are characterized by wide histologic variability. The unique characteristics of tenosynovial giant cell tumors should be always kept in mind, when a diagnosis of a pharyngeal mass is considered to avoid irreversible mistakes.

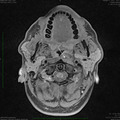

## 1 CASE PRESENTATION

**FIGURE 1:**
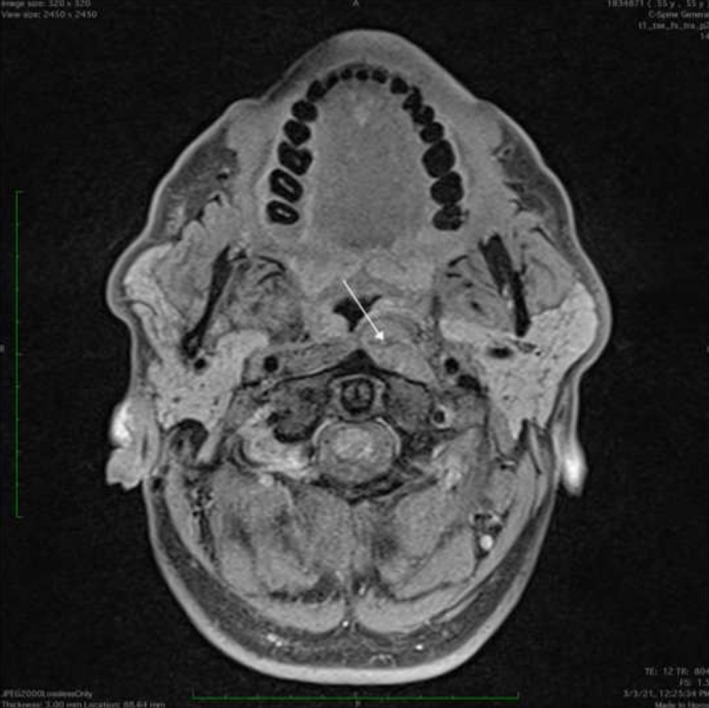
Axial MRI T1 sequence suggestive of a mass in posterior pharyngeal space. (arrow)

**FIGURE 2:**
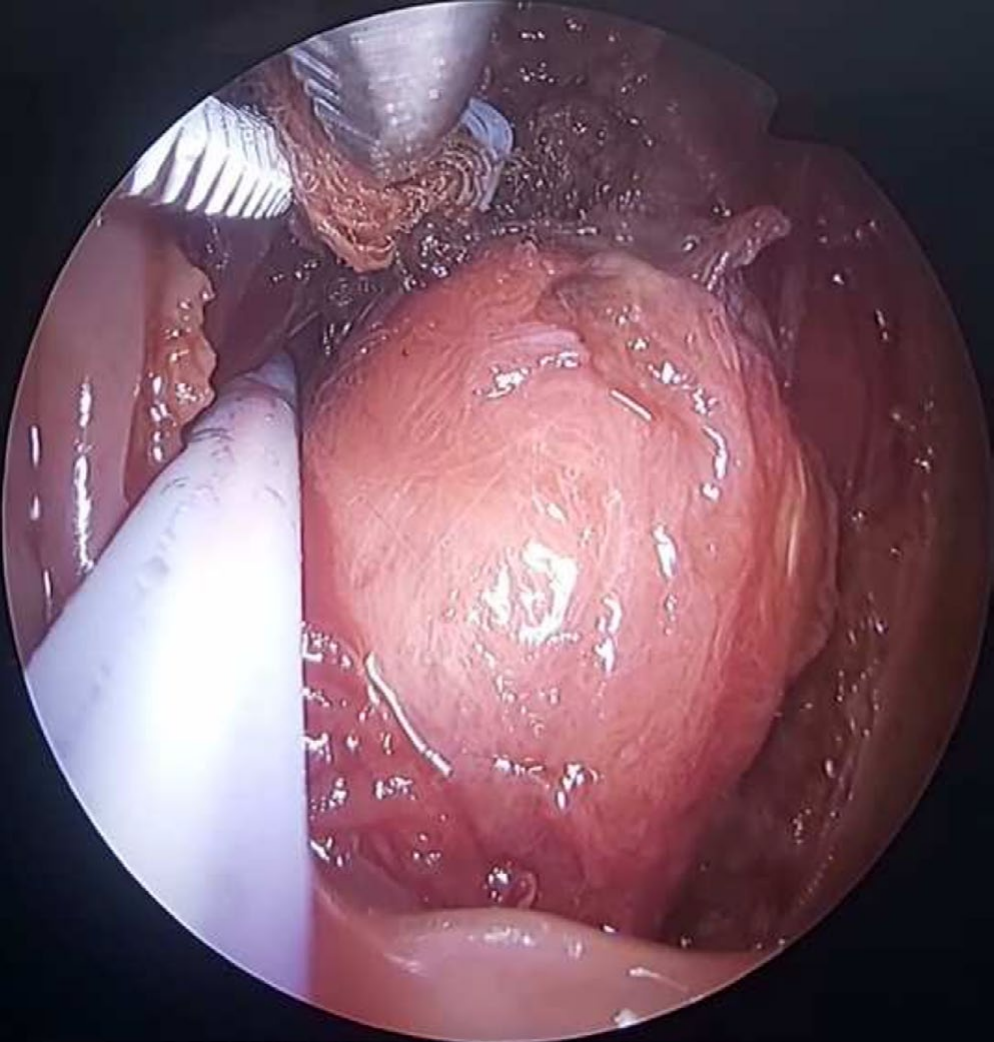
Intraoperative view of the mass and use of Doppler to identify internal carotid artery

**FIGURE 3:**
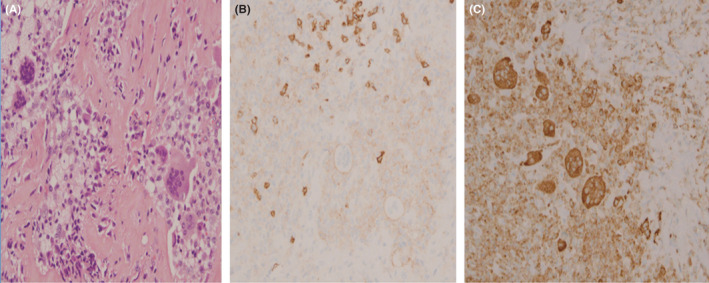
(A) Histologic examination of the tumor. Formations of numerous foam cells. Hematoxylin and eosin (H&E) staining, original magnification X20. (B) Typical mononuclear histiocytic cells and osteoclastic multinucleated giant cells, positive for CD68 magnification X20 (C) Typical mononuclear histiocytic cells and osteoclastic multinucleated giant cells, positive for LCA Immunohistochemistry, original magnification X20

A 55‐year‐old man presented with the chief complaint of dysphagia and sore throat. Oral examination revealed a firm nodular mass in the midline of the pharyngeal wall. Magnetic resonance imaging (MRI) showed a mass in posterior pharyngeal space, with decreased signal intensity on T1 and T2 sequences, without evidence of bone marrow involvement (Figure 1). The tumor was mobilized under endoscopic assistance and was en‐bloc excised (Figure 2). Doppler was recruited intraoperatively to identify the internal carotid artery. A lobular growth was noted among a vitreous layer of mononuclear mast cells, mixed at diffuse sites with osteoclastic multinucleated giant cells. Immunohistochemistry was suggestive of mononuclear histiocytic cells and osteoclastic multinucleated giant cells positive for CD68 and Anti‐CD45 antibodies (Figure 3). Therefore, a tenosynovial giant cell tumor was diagnosed. A decision for no adjuvant therapy was made, according to common practice concerning other sites. Six months postoperatively, the patient remains asymptomatic and with no evidence of disease.

## 2 DISCUSSION

Tenosynovial giant cell tumors are uncommon neoplasms in the head and neck area, with temporomandibular joint being the most commonly affected region.^1^ Neck, cervical spine, clinoid, and posterior pharyngeal wall involvement is even rarer.^2^

## CONFLICT OF INTEREST

No potential conflict of interest was reported by the authors.

## AUTHOR CONTRIBUTIONS

AP, NT, VC, and CG: study design, writing of the draft, and approval of the manuscript for submission.

## CONSENT

Written informed consent was obtained from the patient for the publication of this clinical image.

## Data Availability

None.
